# Mechanisms of radiotherapy resistance and radiosensitization strategies for esophageal squamous cell carcinoma

**DOI:** 10.1186/s12943-023-01839-2

**Published:** 2023-08-19

**Authors:** Lingbo An, Mingyang Li, Qingge Jia

**Affiliations:** 1 State Key Laboratory of Cancer Biology, Department of Pathology, Xijing Hospital and School of Basic Medicine, Fourth Military Medical University, Xi’an, China; 2 College of Medical Technology, Xi’an Medical University, Xi’an, China; 3 Department of Reproductive Medicine, Xi’an International Medical Center Hospital, Northwest University, Xi’an, China

**Keywords:** ESCC, Cancer stem cells, Radioresistance, Molecular targets, Reversal strategies

## Abstract

Esophageal squamous cell carcinoma (ESCC) is the sixth most common cause of cancer-related mortality worldwide, with more than half of them occurred in China. Radiotherapy (RT) has been widely used for treating ESCC. However, radiation-induced DNA damage response (DDR) can promote the release of cytokines and chemokines, and triggers inflammatory reactions and changes in the tumor microenvironment (TME), thereby inhibiting the immune function and causing the invasion and metastasis of ESCC. Radioresistance is the major cause of disease progression and mortality in cancer, and it is associated with heterogeneity. Therefore, a better understanding of the radioresistance mechanisms may generate more reversal strategies to improve the cure rates and survival periods of ESCC patients. We mainly summarized the possible mechanisms of radioresistance in order to reveal new targets for ESCC therapy. Then we summarized and compared the current strategies to reverse radioresistance.

## Introduction

Esophageal cancer is principally comprised of two epidemiologically and pathologically distinct diseases, esophageal squamous cell carcinoma (ESCC) and esophageal adenocarcinoma (EAC), of which ESCC accounts for approximately 90% [1]. ESCC is the sixth most common cause of cancer-related mortality worldwide, with more than half of cases occurring in China [2]. ESCC is an aggressive cancer with rapid growth and a high lymph node metastasis rate, and it commonly affects the upper two-thirds of the esophagus [3]. Dysphagia and cervical lymph node enlargement are not exhibited until the cancer has already become advanced [4], which has a low 5-year survival, and the prognosis is extremely poor.

Currently, radiotherapy (RT) and definitive chemoradiotherapy (CRT) have been used for ESCC patients who are locally advanced (including resectable and unresectable), refuse or are unfit for surgery. RT can promote the release of new tumor antigens, calreticulin, and heat shock protein [5], and increased expression of major histocompatibility complex I (MHC I) can promote dendritic cell (DC) maturation and infiltration into tumor cells to activate the antitumor immune response [6]. Nevertheless, radiation-induced DDR can promote the release of cytokines and chemokines [7] and trigger inflammatory reactions and changes in the tumor microenvironment (TME). There are many immunosuppressive cells in the TME, including cancer-associated fibroblasts (CAFs), macrophages, myeloid-derived suppressor cells (MDSCs), and other stromal cells [8]. A large number of immature MDSCs can inhibit the immune function of T cell through direct intercellular interactions or secrete cytokines, promoting the invasion and metastasis of tumors. Moreover, radiation activates the autophagy signaling pathway that leads to cell death.

In ESCC, radioresistance has remained a significant obstacle to improving the efficacy of RT. Therefore, we mainly summarized the possible mechanisms of radioresistance, including cancer stem cells (CSCs), enhancing DNA damage repair capacity, enhanced ability to scavenge ROS, epithelial-mesenchymal transition (EMT) and abnormal regulation of programmed cell death, to reveal new targets for ESCC therapy. Then, according to the intracellular and extracellular pathways of ESCC radioresistance, we comprehensively summarized the published strategies to reverse ESCC radioresistance and compared and discussed different treatment strategies of the same type of regimen.

## Cellular regulation mechanisms of radioresistance

### Cancer stem cells

CSCs are a small population of cancer cells with properties such as self-renewal, multidirectional differentiation, unlimited proliferation, and high tumorigenicity [9]. Normal stem cells are now considered the primary source of CSCs and tumor-initiating cells because mutations in these cells can lead to an imbalance between self-renewal and differentiation, causing local recurrence, metastasis, and decreased sensitivity to RT. Tumor treatment of esophageal cancer may start at any stage of the stem cell proliferation process, and the degree of tumor differentiation is positively correlated with the stage of stem cell proliferation and differentiation from which the tumor originates. Any obstruction encountered by stem cells during proliferation and division may lead to cell cycle arrest. Tribbles pseudokinase 3 (TRIB3), a mitosis inhibitor, inhibits the ubiquitination and degradation of TAZ by binding to TAZ, thereby promoting the CSC properties and radioresistance of ESCC [10]. Similar to other CSCs, esophageal cancer stem cells segregate different subpopulations of cancer cells according to their specific markers and are involved in promoting esophageal tumorigenesis through renewal and repair. Our laboratory first found that ribosomal S6 protein kinase 4 (RSK4) is highly expressed in ESCC CSCs, activates the β-catenin pathway through direct phosphorylation of GSK-3β at Ser9 and that RSK4 is a direct transcriptional target of ΔNp63α; thus, the ΔNp63α/RSK4/GSK-3β axis is key to driving CSC characterization and resistance to RT in ESCC [11]. In response to these findings, we designed and synthesized, for the first time, a series of 1,4-dihydro-2 H-pyrimido[4,5-d] [1, 3]oxazin-2-ones derivatives, which are novel RSK4 inhibitors that could become effective therapeutic agents for improving the radiosensitivity of ESCC in the future [12]. TWIST-related protein 1 (TWIST1), a transcription factor (TF) of epithelial-mesenchymal transition (EMT), induces the CSC phenotype and amplifies it by coordinating EMT; enhances N-cadherin, occludin, vimentin and ZEB2 expression; promotes the expression of BMI1; induces proliferation and invasion of ESCC cells; and promotes long-term proliferation and differentiation inhibition of ESCC stem cells by increasing the expression levels of stemness TFs KLF4 and SOX2 in KYSE-30 cells [13]. KLF4, as an antiproliferative factor in differentiated epithelium, knockdown significantly reduces TWIST1-induced metastatic activity in vitro and in vivo and decreases tumor initiation capacity [13]. The TWIST1-JAGGED1-NOTCH-KLF4 axis induces stem cell-like features and metastasis, where KLF4 and BMI1 contribute to TWIST1-induced tumor-initiating capacity, and knockdown of BMI1 expression leads to a reduction in the size and number of tumor spheroids [14]. Cluster of differentiation (CD), a class of proteins present on the cell membrane, such as CD14 [15], CD44 [16], and CD47/CD133 [17], can also serve as a surface marker of ESCC stem cells and inhibit the maintenance of tumor stemness by suppressing its expression, making it an independent prognostic factor and a promising therapeutic target for ESCC stem cells.

### Enhanced DNA damage repair capacity

IR causes exogenous damage to DNA, including mismatches, base isomerization, and DNA strand breaks. The PI3 kinase (PI3K) family plays an important role in DNA damage repair, and includes ATM- and Rad3-related kinase (ATR), ataxia-telangiectasia mutated (ATM), and DNA-dependent protein kinase (DNA-PK). Phosphorylation of FMS-related tyrosine kinase 3 (Flt-3) receptor increased significantly by FMS-related tyrosine kinase 3 ligand (FL) after irradiation (IR), further activating the PI3K/AKT/BAD signaling pathway, promoting clone formation and enhancing DDR and inhibiting IR-induced ESCC apoptosis through upregulation of p-Bad anti-apoptotic protein (Ser136 protein) [18]. IR-induced exosomal high-mobility group box 1 (HMGB1) acts synergistically with proteins Bax and Bcl2 to reduce apoptosis through the PI3K/AKT/FOXO3A signaling pathway and participates in IR-induced DDR through γH2AX [19, 20]. WISP1 is a downstream target gene of the Wnt/β-catenin pathway, GSK3β is a key enzyme that inhibits activation of the Wnt/β-catenin pathway. Thus, WISP1 promotes the phosphorylation and inactivation of GSK3β through activation of Akt and mediates radioresistance through activation of anti-apoptotic PI3K kinases [21].

ATM/ATR-dependent phosphorylation of the tumor suppressor BRCA1 occurs at Ser1423 and Ser1524 after IR, influencing G1/S cell cycle progression after DNA damage [22]. ATR and BRCA1 are key regulators of radiation-induced ERK1/2 signaling, which activates the G2 checkpoint. ERK1/2 signaling is associated with upregulation of the transcriptional levels of genes involved in DNA repair. MEK1/2 activates its only downstream target ERK kinase through phosphorylation, thus inhibiting the apoptosis of irradiated cells. Thus, the human chemokine CXCL1 can exert DNA repair by activating the Ras/Raf/MEK/ERK signaling pathway [23]. Intra-S phase cell cycle arrest is initiated by activation of downstream Chk1 by ATR or downstream Chk2, p53 and other proteins by ATM. The irreversible state of cell cycle arrest may further lead to cellular senescence. p53, a key cell cycle regulator protein, activates the cyclin dependent kinase (CDK) inhibitors p21 and p16, both of which are elevated, and Src signaling is diminished, resulting in a lack of CD59 in the cell, which significantly induces ESCC cellular senescence after ionizing radiation [24]. DNA-PK regulates the G1/S checkpoint response to DNA double-strand breaks (DSBs), which are repaired primarily through DNA-PK and Ku-regulated non-homologous end joining (NHEJ) [25]. ATM has been shown to phosphorylate and activate Ku in response to DNA damage [26]. Recent studies have confirmed that RAD18 overexpression increases ESCC radioresistance by upregulating DNA-PK levels [27].

In addition, overexpression of the TF NFE2L3 activates the IL-6-STAT3 pathway, which in turn increases radioresistance [28]; Janus kinase (JAK) is hyperphosphorylated and activated by radiation, creating docking sites for downstream bridging and effector STAT generation. HMBG1 also regulates DNA damage through the MAPK pathway [29]. Therefore, inhibition of the above pathways may be promising strategy to improve the efficacy of RT for ESCC.

### Enhanced ability to scavenge ROS

Radiation potentiates lipolysis to produce oxygen species (ROS) and induce protein carbonylation, which is irreversible oxidative protein damage [30]. Nuclear factor (erythroid-derived 2)-like 2 (Nrf2) is the main mediator of cellular adaptation to oxidative stress. When exposed to oxidative or electrophilic stress, Nrf2 combines with antioxidant response elements (ARE) within the promoter region of target genes and activates related antioxidant molecules, such as NADPH, heme oxygenase-1 (HO1), Nrf2-driven antioxidant molecules NAD(P)H: quinine oxidoreductase-1 (NQO1), etc., thereby defending cancer cells against oxidative damage and enhancing tumor radioresistance [31]. The caspase-8 mutant binds to mTOR, phosphorylates SQSTM1 at Ser349, promotes the interaction between SQSTM1 and Keap1, and reduces the degradation of the Nrf2 protein. In turn, the Nrf2 inhibitor reduces the effect of the caspase-8 mutant on oxidative stress. Thus, the caspase-8 mutant can be protected from oxidative stress through the mTOR/SQSTM1/Keap1/Nrf2 axis to gain protection against oxidative stress [32]. Peroxiredoxin 6, a member of the peroxidase superfamily, functions to eliminate ROS and reduce apoptosis in irradiated cells, suggesting a negative role in RT [33]. In contrast, the chemokine CXCL1 increases ROS levels by inhibiting ROS scavenging enzymes, mainly superoxide dismutase and glutathione peroxidase, which in turn enhances DNA damage repair [23]. Hypoxia-stimulated ROS promotion of elevated tumor malignancy has been extensively studied. Insulin-like growth factor binding protein 3 (IGFBP3) is a hypoxia-inducible gene that regulates apoptosis and EMT in ESCC cells. IGFBP3 cooperates with hypoxia by inhibiting ROS production in an IGF independent fashion [34].

### Epithelial-mesenchymal transition (EMT)

Epithelial to mesenchymal transition (EMT) is the transformation of epithelial cells to mesenchymal phenotype cells [35]. The most significant events of EMT are the downregulation of E-cadherin, the upregulation of the TFs slug and snail and the induction of vimentin and N-cadherin expression, which facilitate the metastasis and spread of cancer cells [36]. The TGF-β family is an important inducer in the EMT program, and high levels of TGF-β act on target cells by pooling with specific receptors, leading to downstream phosphorylation of Smad2 and Smad3 [37]. Phosphorylated Smad2/3 is increased in stable cell lines with high cell division cycle-associated 7 (CDCA7) overexpression, and Smad4 is recruited to form the Smad complex, which binds to and inhibits the expression of the promoter sequence encoding the E-cadherin gene, promoting the EMT process of ESCC [37]. Hypoxic [38] or cancer-associated fibroblasts (CAFs) [39] mediate radioresistance in ESCC by inducing TGF-β activation and paracrine signaling and promoting the expression of EMT markers such as slug, snail, and Zeb1. The TF of the EMT induced EMT program stimulates the acquisition of tumor-initiating CSCs by regulating stem cell markers. Twist, a gene associated with EMT, is also positively regulated by PI3K/AKT signaling [40]. PI3K/AKT activation promotes phosphorylation of GSK-3β, which inhibits snail ubiquitination and degradation and promotes snail-induced EMT in vitro [41]. Regulatory T cells (Tregs), epiplakin1 [42] and neuropilin and tolloid-like 2 (NETO2) [43] reduce the radiosensitivity of ESCC by activating the EMT and PI3K/AKT pathways. Non-coding ribonucleic acids have been considered important factors in regulating the development of EMT by multiple regulatory mechanisms. Recent studies have reported that circVRK1 [44] modulates the effects of RT by regulating the PTEN/PI3K/AKT signaling pathway. miR-301 A [45], miR-1275 [46] and lncRNA LINC00675 [47] inhibit ESCC genesis and EMT activation by targeting Wnt/β-catenin signaling. Moreover, lncRNA MEG3 inhibits EMT through the GSK-3β/snail signaling pathway [48].

### Abnormal regulation of programmed cell death

Programmed cell death includes apoptosis, autophagy, ferroptosis, necroptosis, pyroptosis, of which autophagy and ferroptosis have recently been found to regulate immunosuppressive TME in ESCC. Autophagy is a conserved catabolic process that promotes the recycling of intracellular components, degrades damaged proteins and organelles, and then selectively reabsorbs them through autophagic vesicle nucleation and autophagic lysosome formation. However, under adverse conditions such as nutritional deficiency, oxidative stress and radiation, tumor cells will select autophagy as a survival mechanism. Therefore, tumors are more sensitive to autophagy inhibition than normal tissues. Autophagy-mediated tumor survival is mainly attributed to (i) dysregulation of mitochondrial autophagic clearance and regulation of oxidative stress promoting the EMT response to GSK-β and (ii) subsequent triggering of genomic instability. AMP-activated protein kinase (AMPK) promotes autophagic vesicle maturation and activates autophagy by assembling the Unc51-like kinase 1 (ULK1) complex, while mammalian target of rapamycin complex 1 (mTORC1) does the opposite [49]. For example, human butyrophilin subfamily 3 member A1 (BTN3A1), which is structurally related to the B7 costimulatory molecule, induces autophagy by promoting ULK1 phosphorylation at Ser555 and confers radioresistance to ESCC, suggesting that BTN3A1 may be a new therapeutic target [50]. The CDK4/6 inhibitor palbociclib activates the autophagy pathway by inhibiting mTOR to reverse radioresistance [51]. In contrast, many signaling pathways regulate mTORC1 activity, including the AMPK/mTOR pathway [52], p53 pathway [53] and PI3K/AKT/mTOR pathway [54]. For example, the ubiquitin-specific peptidase 8 (USP8) inhibitor DUB-IN-1 can inhibit ESCC cell growth by stimulating autophagy through p53-dependent adenosine 5’-monophosphate (AMP)-activated protein kinase (AMPK) [55]. Allicin promotes cellular autophagy and ferroptosis in ESCC through activation of AMPK/mTOR [56]. Downregulation of fibulin-4 expression inhibits autophagy and promotes the sensitivity of ESCC cells to apatinib through activation of the Akt/mTOR signaling pathway [54].

Nrf2 is also a potential regulator of iron homeostasis and redox balance in cancer cells [57]. Ferroptosis is a regulated cell death caused by iron-dependent accumulation of lipid peroxides in excess, and inhibition of ferroptosis results in reduced radiosensitivity of ESCC cells. Patients with high Nrf2 expression had significantly shorter overall survival (OS) and progression-free survival (PFS). Nrf2 inhibits RT-induced ferroptosis mainly by directly binding to the promoter region of solute carrier family 7 member 11 (SLC7A11), thereby reducing ROS and lipid peroxidation levels, and this effect can be eliminated by ferroptosis inducers, but the exact mechanism of this finding needs to be further explored [58]. Glutathione peroxidase 4 is recognized as a negative regulator of ferroptosis, and cysteine is the rate-limiting amino acid for glutathione production. Acetyl-CoA acetyltransferase 2 (ACAT2) overexpression confers radioresistance by inhibiting ESCC ferroptosis, and after knockdown of ACAT2, cells can produce higher ROS after IR and increase radiosensitization by reducing glutathione peroxidase 4 levels [59].

**Fig. 1:**
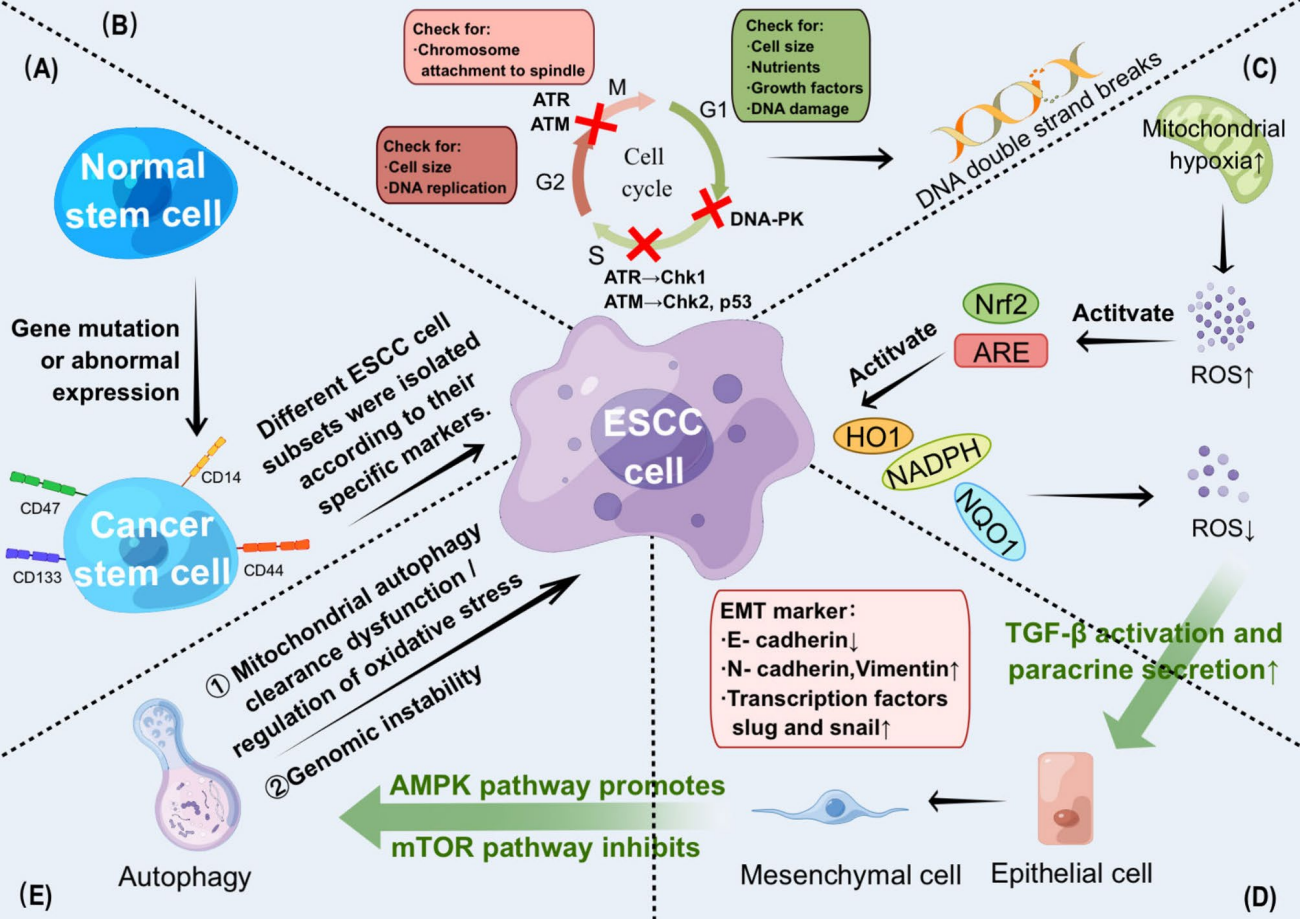
Cellular regulation mechanisms of radioresistance. (A) Normal stem cells are transformed into cancer stem cells through gene mutation or abnormal expression, and different ESCC cell subsets are separated according to the specific markers on them. (B) In the cell cycle, the G1 checkpoint is responsible for the cell size, nutrients, growth factors and DNA damage; the G2 checkpoint is responsible for the cell size and DNA replication; the M checkpoint is responsible for the chromosome attachment to spindle. DNA-PK regulates the response of G1/S checkpoint to DNA double strand breaks (DSB); ATR activates downstream Chk1 or ATM activates downstream Chk2, p53 and other protein, which starts the arrest in S-phase cell cycle; ATM and ATR induced G2/M phase arrest. (C) Mitochondrial hypoxia leads to the increase of ROS release, which promotes the nuclear factor (erythroid-derived 2)-like 2 (Nrf2) to combine with the antioxidant response element (ARE) in the promoter region of the target gene, and activates related antioxidant molecules, such as NADPH, heme oxygenase-1(HO1), and NADPH:quinone oxidoreductase-1(NQO1) driven by Nrf2. So as to reduce or eliminate the generation of ROS, prevent cancer cell damage caused by redox, and enhance the radioresistance of tumors. (D) Hypoxia or tumor-related fibroblasts promote the expression of EMT markers such as slug, snail and Zeb1 by inducing TGF-β activation and paracrine, and mediate the radioresistance mechanism of ESCC. (E) Autophagy-mediated tumor survival is mainly attributed to: (i) clearing dysfunction through mitochondrial autophagy and regulating oxidative stress to promote EMT’s response to GSK-β. (ii) Genomic instability caused subsequently. AMP-activated protein kinase (AMPK) activates autophagy, while mammalian target of rapamycin complex 1 (mTORC1) does the opposite

In addition to protecting cells from ROS damage, Nrf2 appears to play a direct role in cell growth control. Xia et al. [60] reported that Nrf2 targets Ca^2+^/calmodulin-dependent protein kinase II α (CaMKIIα) by inhibiting mTOR and p62 phosphorylation, activating Beclin 1 and subsequently activating autophagy to promote ESCC radioresistance. Therefore, inhibition of autophagy may become a key in the fight against cancer. Lu et al. [61] demonstrated by targeting Nrf2 in ESCC that sulforaphane (SFN), a natural anticancer compound, induced apoptosis and inhibited autophagy, thereby improving anticancer efficiency in ESCC patients. SFN also inhibited autophagy via mTOR/TFE3 and induced exosome-mediated cellular senescence [62]. Among non-coding ribonucleic acids, ciRS-7 inhibits ESCC cell autophagy by targeting epidermal growth factor receptor (EGFR) signaling as a miR-1299 sponge [63]. MiR-126 inhibits ESCC cell autophagy by targeting the STAT3 signaling pathway [64]. Recent studies have identified METTL1 and WDR4, components of the m^7^G methyltransferase complex, as negative regulators of MTORC1-mediated autophagy in ESCC; thus, METTL1 and its downstream signaling axis, the RPTOR/ULK1/autophagy pathway, may become novel regulators of autophagy in ESCC cells [65]. We summarize the mechanism of radioresistance within ESCC cells in Fig. 1.

## Tumor microenvironment (TME) and radioresistance

### Cancer-associated fibroblasts

Fibroblasts are quiescent cells that reside in connective tissue and are derived from mesenchyme, which are activated in response to injury or inflammatory conditions in the body. When the body has an autoimmune disease or develops cancer, fibroblasts begin to repair the tissue continuously, and this epigenetic modification maintains the wound healing state, leading to the emergence of a subpopulation of hyperactivated fibroblasts. This subpopulation of hyperactivated fibroblasts with elongated morphology, enhanced proliferation and resistance to apoptosis but lacking mutations, present within or near the cancer mass, is called CAF [66]. CAFs conferred direct radioresistance or predicted tumor radioresponse and therefore should be used as a major target for improving tumor radiosensitivity in esophageal carcinoma [67].

CAF-derived tumor-promoting factors and exosomes promote ESCC progression, i.e., EMT, which may confer radioresistance and the ability of cancer cells to invade distant sites [68]. Proteins, inflammatory cytokines, growth factors and non-coding RNAs derived from CAF exosomes interact with ESCC cells. For example, CAF-secreted plasminogen activator inhibitor-1 (PAI-1) [69] and TGF-β [70] induce the migration and invasion of ESCC cells through the AKT and ERK1/2 signaling pathways or promote the radioresistance of ESCC through CAF-derived exosomal miR-3656 via the ACAP2/PI3K-AKT pathway [71]. As we mentioned previously, the enhanced capacity of DDR is not conducive to radiation, which can cause lethal damage to cancer cells. CXCL1 secreted by CAFs mediates radioresistance by inhibiting the ROS-scavenging enzyme superoxide dismutase 1, causing ROS accumulation and thus enhancing DDR [23]. Recent studies have reported that CXCL1 secreted by ESCC cancer cells stimulates the conversion of CAFs toward its subtype of inflammatory CAFs (iCAFs), further promoting cancer progression [72]. LncRNA DNM3OS promotes DDR by increasing the expression of DNA repair proteins; thus, CAFs significantly promote lncRNA DNM3OS expression in esophageal cancer cells by activating the PDGFβ/PDGFβR/FOXO1 pathway as a way to confer radioresistance [73]. Moreover, the HGF, IGF, and TGF-β secreted by CAFs also promote deposition and remodeling of extracellular matrix proteins that lead to leakage of normal blood vessels and promote angiogenesis of tumor cells. As a receptor for HGF, c-Met is a commonly overexpressed oncogene in ESCC. c-Met activation facilitates ESCC invasion and proliferation. A c-Met inhibitor, BPI-9016 M, exerts radiosensitization of ESCC cells by reducing phosphorylation of ATR and ATM, upregulating apoptosis-related molecules and inhibiting homologous recombination (HR) [74]. Small interfering RNA (siRNA) is currently used mainly to regulate the expression of tumor suppressors or target oncogene therapy. Effective inhibition of the IGF-1 receptor with siRNA gene silencing technology combined with RT can help enhance the radiosensitivity of ESCC cancer cells, but the exact mechanism has not been elucidated [75]. Therefore, targeting EGFR or HGF/Met signaling pathways may help to block ESCC invasion and spread.

Excitingly, CAFs not only directly contact ESCC cells but also interact with ESCC cell metabolites. CAFs provide cancer cells with amino acids, fatty acids, glucose, phospholipids and glycerides, which are essential for ESCC growth; cancer cells secrete hydrogen peroxide, which increases oxidative stress in CAFs and induces a transformation of the metabolic environment of CAFs from oxidative phosphorylation to aerobic glycosylation that further provides lactate and pyruvate to cancer cells [76]. Downregulation of LncRNA-CAF, FLJ22447 under hypoxic conditions was significantly associated with hypoxia-inducible factor (HIF) 1 A activation of VEGF high expression, which plays a role in promoting angiogenesis and tumor metastasis [77]. A recent study of RT combined with baicalein clearly showed that modulating cellular metabolism and inhibiting glycolysis by targeting HIF-1 A significantly affects the radiosensitivity of ESCC. Thus, CAFs could further promote radioresistance in ESCC by crosstalk with ESCC cancer cells through cellular metabolites [78]. Finally, CAFs exert immunosuppressive effects by crosstalk with tumor-associated macrophages (TAMs), which shifts the behavior of TAMs toward tumor promoters, or by inhibiting T-cell functions.

### Tumor-associated macrophages

TAMs, as well as CAFs, can produce various protumor factors, including the growth factors VEGF, CCL2, CCL5, CSF-1 and other proteins. Then, circulating monocytes and macrophages are recruited into the TME, including within or near the tumor. For example, CCL1 promotes ESCC proliferation through the CCR8-mediated AKT/mTOR pathway [79].

Similar to macrophages, TAMs are divided into M1 and M2 subtypes. M1-like TAMs exert proinflammatory and tumor suppressive effects by activating cytokines in cytotoxic T cells through phagocytosis and secretory effects or by presenting tumor antigens to activate cytotoxic T cells [80]. M1-like TAMs are the main source of IL-23, through which ESCC acquires some of its CSC properties, and the radioresistance of human ESCC xenografts depends mainly on IL-23-mediated activation of the Wnt/Notch pathway, which causes cells to stagnate in the G0/G1 phase and finally reduces radiation-induced apoptosis [81]. Heat shock factor (HSF)-1 supports macrophage production by inducing increased expression of HIF-1, which may be essential in the formation of M2-like TAMs in response to inflammatory stress [82], and the formed M2-like TAMs mainly exert anti-inflammatory and tumor-promoting effects [83]; therefore, macrophages recruited into the TME are thought to be polarized from the M1 subtype to the M2 subtype [84], and high doses of radiation are one of the factors that promote this polarization. Radiation-induced dioxygenase 12-lipoxygenase (12-LOX) overexpression in ESCC tissues upregulates CCL5 levels, which attracts THP-1-derived macrophages and promotes their polarization to the M2 subtype and thereby inhibiting radiation-induced apoptosis [85]. Similarly, the expression of sialic acid binding Ig-like lectin 9 (SIGLEC9) on macrophages in ESCC tissues after RT increases the levels of M2-like TAM markers. Mucin 1 (MUC1) is a ferroptosis suppressor that activates SIGLEC9 transcription and synergistically induces M2-like TAM polarization, thereby promoting radioresistance in ESCC [86]. Therefore, inhibiting M2-like TAM polarization or inducing the conversion of M2-like TAMs to the M1 subtype may be an effective option to reverse radioresistance in ESCC. It was confirmed that knockdown of LINC01004 or SPI1 could specifically inhibit SIGLEC9, induce TAM reprogramming and eventually convert to the M1 subtype, thus alleviating immunosuppression and radioresistance in ESCC [86]. TAMs also induce EMT by secreting EGF, TNF-α and TGF-β, promoting the migration and invasion of ESCC, and their accumulation in the microenvironment creates an immunosuppressive and tumor-supportive environment.

In addition, as in many malignancies, ESCC can evade immune destruction by developing mechanisms that the immune system regularly uses for self-regulation, the most extensively studied of which is programmed death protein 1 (PD-1). PD-1 inhibits the T-cell-mediated immune response by binding to its ligands PD-L1 or PD-L2. Yang et al. [87] demonstrated that M2 polarization increased PD-L2 expression in TAMs and then activated the PD-1 signaling pathway to promote tumor proliferation; blocking the CCL2-CCR2 axis impeded TAM recruitment, thereby enhancing the anti-ESCC efficacy of CD8 T cells. Naomichi Koga et al. [88] reported that signal regulatory protein alpha (SIRPα) may induce immunosuppression by inhibiting TAM phagocytosis and that coexpression of SIRPα and PD-L1 is significantly associated with ESCC infiltration and has a worse prognosis than patients expressing the proteins alone or expressing neither. Therefore, TAM combined with PD-1 inhibitors may provide a new idea for reversing radioresistance in ESCC.

### Other immunosuppressive cells

A specific type of immune cell is present in all tissues of the body and is called Tregs. It promotes immune homeostasis through highly specialized tissue-specific pathways, coordinates the suppression of excessive immune activation to suppress autoimmunity, and maintains immune homeostasis. Treg recruitment was associated with the chemokines CCL2, CCL17 and CCL22 secreted by tumor cells and TAMs, which significantly induced Treg migration [89]. Moreover, Tregs are capable of secreting immunosuppressive mediators, including cytokines such as IL-1, IL-6, IL-10, IL-35 and TGF-β and small molecules such as adenosine. IL-32 expression in ESCC and Treg infiltration play an important synergistic role in tumor growth and invasion, and multifactorial analysis showed that both were independent risk factors [90]. Although there are currently no studies of Treg-associated ESCC radioresistance, the role played by Tregs in head and neck squamous cell carcinoma (HNSCC) radioresistance has been reported. Inhibition of the STAT3 pathway and CCR6-CCL20 axis prevented Treg infiltration and inhibited tumor growth, thereby enhancing the radiosensitivity of HNSCC, and activating dendritic cells which reduced the growth of tumor cells that were resistant to radiotherapy [91–93]. In view of the genetic similarity between HNSCC and ESCC, the radioresistance of Tregs in HNSCC may have similar effects in ESCC.

**Fig. 2:**
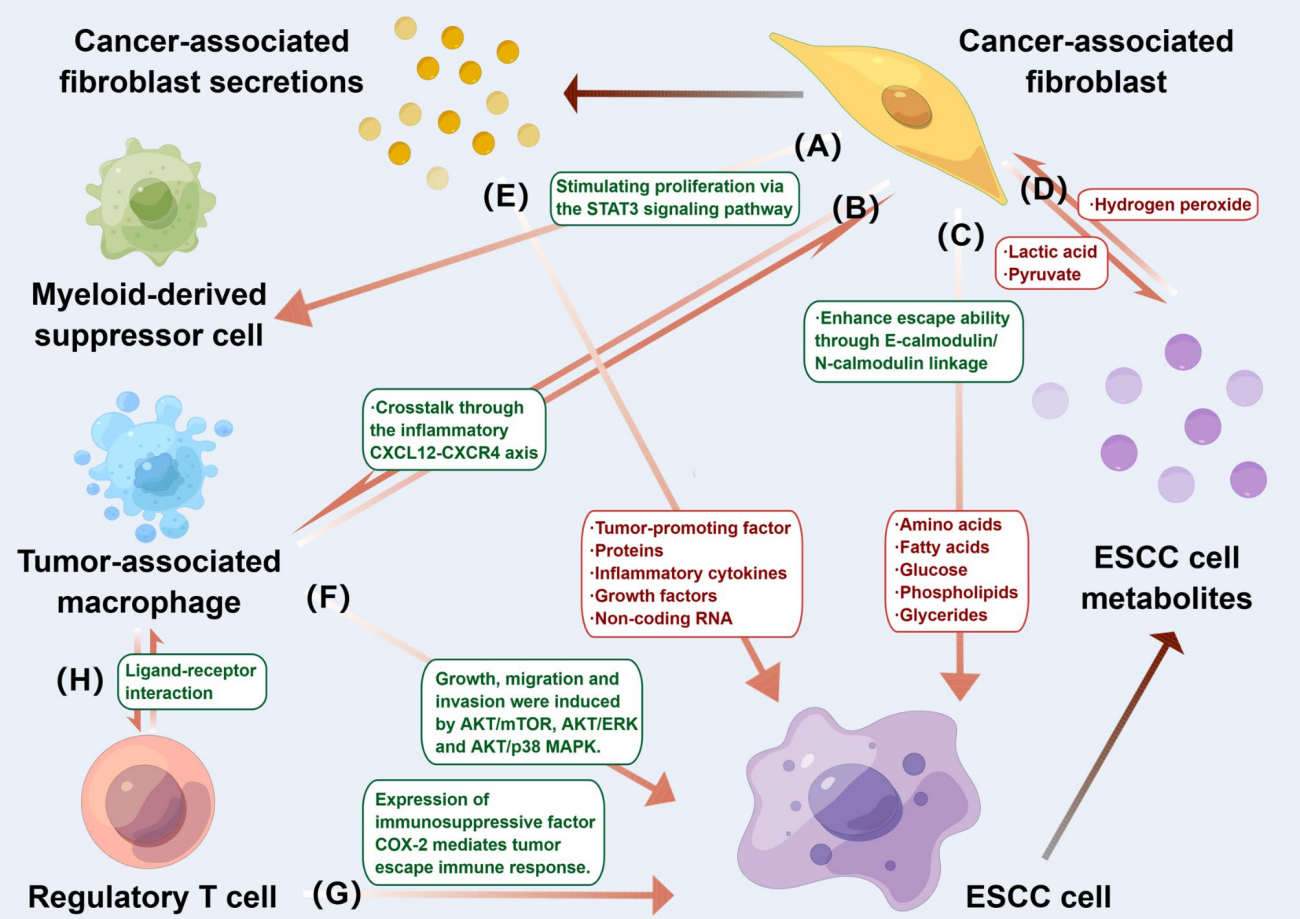
Tumor microenvironment (TME) and radioresistance. (A) CAF induces monocyte MDSC production through STAT3 signaling activated by IL-6/exosomal miR-21. (B) CAF crosstalk with TAM through the inflammatory CXCL12-CXCR4 axis. (C) CAF provides cancer cells with amino acids, fatty acids, glucose, phospholipids and glycerides that are essential for ESCC growth; and enhances escape through E-cadherin/N cadherin linkage enhances escape. (D) Cancer cells secrete hydrogen peroxide, which increases oxidative stress in CAF and induces a shift in the metabolic environment of CAF from oxidative phosphorylation to aerobic glycosylation, further providing cancer cells with lactate and pyruvate. (E) CAF secretions, including pro-tumor factors, proteins, inflammatory factors, growth factors, and non-coding RNAs induce migration and invasion of ESCC cells. (F) TAM provides ESCC cells with a variety of amino acids through AKT/mTOR, AKT/ERK and AKT/p38 MAPK induce ESCC cell growth, migration and invasion. (G,H) Regulatory T cell (Treg) mediate ESCC evasion of immune responses through expression of the immunosuppressive factor COX-2, whose interaction with TAM via ligand-receptor interactions may contribute to the immunosuppressed state and disease progression

MDSCs are important immunosuppressive cells in the TME that can largely impair the cytotoxic and killing functions of T cells and NK cells in the antitumor process and promote ESCC development. CAFs induces monocytic MDSC production through STAT3 signaling activated by IL-6/exosomal miR-21 [94]. Neural precursor cell expressed developmentally downregulated 9 (NEDD9) acts as a marker of ESCC, regulates CXCL8 through the ERK pathway, recruits MDSCs into tumor tissue, and maintains ESCC cell stemness through the Notch pathway, which in turn enhances the radioresistance of cancer cells [95]. Furthermore, miR-26b-5p was upregulated in dying human ESCC cells after IR and induced MDSC activation by targeting the PI3K/Akt pathway, creating an immunosuppressive microenvironment that could inhibit IR-induced cancer cell death and create opportunities for cancer invasion [96]. We summarize the mechanism of radioresistance in the ESCC microenvironment in Fig. 2.

## Reversal strategy of ESCC radioresistance

### Combining immune checkpoint inhibitors and radiotherapy

The most extensive studies related to the reversal of ESCC radioresistance are currently targeting PD-1. Table 1 summarizes the clinical trials targeting PD-1 inhibitors in combination with RT/CRT for ESCC that are enrolling. As previously described, PD-1 and its ligands (PD-L1 and PD-L2) couple to inhibit the T-cell-mediated immune response and promote tumor invasion and spread. PD-1 inhibitors reactivate T cells in an IFN-γ-dependent manner to promote G2/M phase block, apoptosis and DNA damage [97], further enhancing the host immune response, inhibiting cancer cell immune escape, and improving radiosensitivity.

**Table 1:** Clinical trial of targeted PD-1 inhibitor combined with RT/CRT for ESCC being recruited

Tumor type	Study cohort	Control cohort I	Phase	Primary endpoints	Registration
ESCC/EAC/GEJC	Pembrolizumab + FP or FOLFOX therapy + RT	Placebo + FP or FOLFOX therapy + RT	III	EFS, OS	NCT04210115
Locally advanced ESCC	Sintilimab + cisplatin + paclitaxel + RT	Cisplatin + paclitaxel + RT	III	OS	NCT05357846
Locally advanced resectable ESCC	Tislelizumab + carboplatin + paclitaxel + RT	Carboplatin + paclitaxel + RT	III/II	pCR, OS	NCT04973306
Locally advanced ESCC	Tislelizumab + cisplatin + paclitaxel + RT + 12 additional cycles of tislelizumab	Tislelizumab + cisplatin + paclitaxel + RT	II	PFS	NCT05520619
Resectable thoracic ESCC	PD-1 inhibitor + cisplatin + albumin-bound paclitaxel	Cisplatin + albumin-bound paclitaxel + RT	II	pCR	NCT05007145
Resectable ESCC	Pembrolizumab + carboplatin + paclitaxel + RT	-	II	pCR	NCT04435197
Oligometastatic ESCC	Tislelizumab + Triprizumab + cisplatin + albumin-bound paclitaxel + RT	-	II	LCR	NCT04821765
Locally advanced resectable ESCC	Toripalimab + carboplatin + paclitaxel + RT	-	II	pCR	NCT05424432
Locally advanced ESCC	Toripalimab + carboplatin + paclitaxel liposome + RT	-	II	pCR	NCT04644250
Metastatic ESCC	Standard chemotherapy + PD-1 inhibitor + RT + capecitabine	Standard chemotherapy + PD-1 inhibitor	II	1-year PFS	NCT05512520
Advanced ESCC	Tislelizumab + cisplatin + albumin-bound paclitaxel + RT	-	II	MPR, pCR	NCT05323890

GEJC: gastroesophageal junction carcinoma; FP therapy: cisplatin + 5-fluorouracil + RT; FOLFOX therapy: oxaliplatin + leucovorin or levoleucovorin + RT; EFS, event-free survival; OS, overall survival time; pCR, pathological complete response rate; PFS, progression-free survival; LCR, locoregional control rate; MPR, major pathological response rate

At the resectable ESCC stage, a phase I trial (PALACE-1) conducted by Li et al. [98] explored the safety of the preoperative anti-PD-1 antibody pembrolizumab in combination with CCRT (PPCT) for ESCC first. Nineteen of 20 patients (95%) received complete preoperative treatment and 18 (90%) underwent surgery with 90% feasibility. Patients with incomplete neoadjuvant therapy developed grade III leukopenia and lymphocytopenia and did not receive the last dose of chemotherapy. The reasons for not receiving surgery were progressive disease due to liver metastases 2 weeks after completion of PPCT in one patient and death due to esophageal hemorrhage while waiting for surgery in another patient. Based on the radiological evaluation after neoadjuvant therapy, complete metabolic response (cMR) was achieved in 6 patients. The median tumor size reduction was 33.3% (SD, 20.4%), and the median maximum standardized uptake value (SUVmax) reduction reached 49.5% (SD, 29.4%). R0 resection was achieved for 17 of the 18 patients (94%) who underwent surgery. This author concludes that pembrolizumab combined with CCRT is safe, does not delay surgery, and induces pathological complete response (pCR) in 55.6% of resected tumors.

At the advanced unresectable ESCC stage, Wei et al. [99] conducted a phase Ib clinical trial of first-line treatment with RT combined with the anti-PD-1 antibody camrelizumab in patients with locally advanced ESCC who were CCRT intolerant or refused CCRT, versus ESCC patients with CCRT alone for dynamic changes in peripheral blood CD8 T-cell function and differentiation comparison. The results showed that in the RT combined with camrelizumab group, camrelizumab effectively bound to PD-1 on CD8 T cells and competed directly with PD-L1 on tumor cells, thereby reducing the suppressive effect of tumor cells on immune cells. Tcm subsets and Tem subsets were increased in PD-1CD8 T cells after RT plus camrelizumab treatment, whereas only Tcm subsets were increased after CCRT treatment, suggesting that RT with camrelizumab treatment may exhibit a stronger CD8 T-cell response than CCRT [99]. Based on the stronger T-cell immune response with RT in combination with camrelizumab than with CCRT, Zhang et al. [100] further explored the safety and feasibility of combining CCRT with camrelizumab as a first-line treatment for patients with locally advanced ESCC. The most common treatment-related grade 3 adverse events in 20 patients included radiation esophagitis (20%) and esophageal fistula (10%). Although serious treatment-related adverse events occurred in 8 patients (40%), no treatment-related deaths were reported and there was no health-related deterioration in quality of life. The OS and PFS times were 8.2–28.5 months and 4.9–28.5 months, respectively. The OS rates at 12 and 24 months were 85.0% and 69.6%, respectively; the PFS rates were 80.0% and 65.0%, respectively. In addition, the PD-1 inhibitor pembrolizumab in combination with GM-CSF and stereotactic body radiotherapy (SBRT) for advanced metastatic ESCC showed significant systemic efficacy, and the mechanism may involve radio sensitization of anti-PD-1 immunotherapy. However, this case was followed by death due to severe pneumonia [101], therefore so we should pay more attention to the safety of the combination therapy in future studies.

### Targeted therapy in combination with radiotherapy

#### Targeted EGFR pathway

EGFR is a member of the receptor tyrosine kinase ErbB family and is indispensable in cell survival, proliferation and differentiation. Since the mutations and overexpression of EGFRs can be observed in esophageal cancer, they can be used as therapeutic targets. Monoclonal antibodies, such as cetuximab and nimotuzumab, hinder cancer progression by recognizing the extracellular region that binds EGFR and blocking the binding of EGF to the receptor. Previous studies have shown that cetuximab can be used safely with CRT in patients with resectable ESCC and to improve clinical remission rates in patients with locally advanced ESCC [102]. In patients with EGFR overexpression, cetuximab not only improves survival but also reduces tumor recurrence and metastasis [103]. However, with the addition of cetuximab to CCRT, skin toxicity becomes a common adverse effect, so it is necessary to pay attention to the toxicity induced by the combination treatment and to reduce the incidence of adverse effects. Compared with cetuximab, nimotuzumab did not have the severe skin and mucosal toxicity associated with other EGFR-targeting antibodies [104]. High-dose nimotuzumab improved survival in patients with esophageal cancer treated with RT [105]. On the basis of CCRT, nimotuzumab was more effective than cetuximab in patients with locally advanced ESCC, with a tendency toward prolonged survival [106]. In addition, radiation can induce autophosphorylation of EGFR proteins and downstream substrates. EGFR tyrosine kinase inhibitors (TKIs) are small chemical inhibitors that target the intracellular portion of the receptor. By preventing intracellular tyrosine kinase activity and obstructing EGFR autophosphorylation, these inhibitors can increase the sensitivity of cells to RT. Icotinib, an orally administered EGFR TKI, significantly inhibited the proliferation of the human epidermoid squamous carcinoma A431 cell line [107]. A phase II clinical trial conducted by Luo et al. [108] showed that icotinib combined with concurrent RT was well tolerated in elderly ESCC patients compared to RT alone, with significantly prolonged 2-year OS and PFS, and patients with EGFR overexpression benefited more from icotinib in combination with RT. Wang et al. [109] further concluded that all patients with advanced ESCC who responded to icotinib showed EGFR overexpression and that remission rates were significantly higher in ESCC patients with high levels of EGFR expression than in those with low to moderate EGFR expression (17.6% vs. 0%). Erlotinib and gefitinib are also specific EGFR TKIs, and once-weekly paclitaxel combined with erlotinib and concurrent RT is expected to be an effective and tolerated regimen for patients with unresectable locally advanced ESCC [110], while gefitinib and concurrent RT are effective and tolerated in elderly ESCC patients [111]. It is important to emphasize that for EGFR-targeted therapy in esophageal cancer, two points must be considered: (i) EGFR-targeted therapy in esophageal cancer resistance due to EGFR-related gene mutations. Activation of the JAK/STAT pathway contributes to gefitinib resistance, and cucurbitacin B (a JAK/STAT signaling inhibitor) can be used in combination with gefitinib to overcome chemoresistance and improve treatment efficacy [112]. (ii) Single-targeted therapy resistance caused by the presence of a compensatory signaling pathway. IGF-1R was found to be compensated by activation after the EGFR/HER2 signaling pathway was inhibited, so the efficacy of receptor TKIs alone was not significant, while the combination of the dual EGFR/HER2 inhibitors lapatinib and gefitinib with the IGF-1R inhibitor linsitinib, inhibited the mutual crosstalk between EGFR/HER2 and IGF-1R, remarkably enhancing ESCC cell cycle arrest and apoptosis [113].

#### Targeted VEGF/VEGFR pathway

Tumor cells and their surrounding stromal cells can secrete VEGF to promote neovascularization and form a unique vascular system within the tumor tissue. Inhibition of VEGF expression ameliorates local hypoxia and improves radiosensitivity [114]. Sunitinib, a highly selective multitargeted receptor TKI, can radiosensitize hypoxic ESCC cells and promote apoptosis in ESCC cells by inhibiting HIF-1α and VEGF upregulation but does not alter their cell cycle distribution [115]. 2-Methoxyestradiol [116] and berberine [117] had similar effects to sunitinib. Unlike sunitinib, zoledronic acid combined with radiation has a radiosensitizing effect on ESCC cells by blocking the cell cycle between S and G2/M phases, leading to increased cell death [118]. Anlotinib selectively inhibits VEGFR and contributes to the inhibition of tumor growth and metastasis, significantly improving PFS and disease control rate (DCR) in patients with advanced ESCC, and is now entering second-line and further-line treatment in ESCC [119]. Cisplatin-based CCRT toxicity is difficult to tolerate in patients with unresectable advanced ESCC, and RT combined with cisplatin and anlotinib or RT combined with paclitaxel and erlotinib can achieve better anti-esophageal cancer outcomes [110, 120]. As a novel selective inhibitor of VEGFR-2, apatinib has previously entered second- or third-line therapy for advanced ESCC. Combining apatinib in ESCC patients treated with RT significantly improves median survival time but does not significantly improve OS [121]. Camrelizumab, a humanized high-affinity IgG4-kappa monoclonal antibody against PD-1, in combination with apatinib may result in mildly toxic durable complete remission in advanced ESCC patients with disease recurrence after receiving RT and counteracts camrelizumab-induced reactive cutaneous capillary endothelial proliferation, which is now in the first-line trial setting [122, 123]. Moreover, the immunosuppressive effects of RT include recruitment of specific immune subpopulations and differentiation of immune subpopulations into a tumor-promoting phenotype [124]. Studies have demonstrated that the antiangiogenic agents sunitinib [125] and imatinib [126] can reduce the number and effectiveness of MDSCs, and bevacizumab can effectively promote the maturation of dendritic cells and reduce immunosuppression [127] but also reduce the recruitment of Tregs [128], thereby inhibiting the quantity and effectiveness of MDSCs. Immunosuppression induced by radiotherapy is expected to show better anticancer effects in ESCC later. In addition, VEGF is associated with the prognosis of ESCC during concurrent RT, and changes in serum VEGF levels (∆VEGF2) after RT are independent influencing factors on OS and PFS in ESCC patients [129].

#### Targeted PI3K/AKT/mTOR pathways

VEGF/VEGFR interactions can trigger multiple signaling pathways, such as the ERK1/2 and PI3K/AKT pathways, leading to tumor cell proliferation, migration and survival. It was demonstrated that knockdown of VEGF expression levels after receiving radiation can activate the NF-kB pathway or PI3K/mTOR signaling pathway [130, 131], which induces DNA damage in cancer cells and contributes to apoptosis, further increasing the lethality of radiation on cancers. Ubiquitin-like with plant homeodomain and ring-finger domains 1 (UHRF1) is a nuclear protein involved in cell growth and is an important link between DNA methylation and histone modifications. In vitro and in vivo experiments showed that inhibition of the PI3K/AKT/mTOR signaling pathway by UHRF1 knockdown inhibited ESCC cell growth and enhanced tumor radiosensitivity, and shUHRF1 binding to radiation significantly increased ESCC cell apoptosis [132]. Similar to UHRF1, miR-34a [133] and miR-519 [134] reversed radioresistance in ESCC cells by inhibiting the PI3K/AKT/mTOR signaling pathway. Treatment with the mTOR inhibitor temsirolimus significantly inhibited the activation of mTOR effectors and reduced ESCC cell proliferation [135]; everolimus significantly inhibited angiotensin II-induced ESCC cell proliferation [136]. However, there are no studies related to the reversal of ESCC radioresistance by everolimus or temsirolimus, which provides a direction for future studies on targeted mTOR inhibitors against ESCC radioresistance.

#### Targeted HGF/c-Met pathway

HGF acts as a cytokine that binds to MET, leading to receptor homodimerization and transphosphorylation of tyrosine residues, resulting in multiple pathways that activate and regulate cell survival, proliferation, differentiation, and angiogenesis. Inhibition of the HGF/c-Met pathway not only reduces the ability of transformed esophageal cells to invade the extracellular matrix but also contributes to IR-induced apoptosis and G2/M phase arrest. Targeted inhibition of the HFG/c-Met signaling pathway has two pathways: one is to prevent HGF from binding to c-Met, and the other is to directly target c-Met, Most of the current studies have focused on the latter. For example, the c-Met inhibitor BPI-9016 M was used to assess its radiosensitizing potential in human ESCC cells in vitro and in vivo, and the results showed that the combination of BPI-9016 M with IR significantly retarded the growth of ESCC tumor xenografts by inhibiting DNA HR repair compared to RT alone [74]. Foretinib is an oral TKI, and compared to foretinib or RT alone, the combination of foretinib with IR significantly enhanced radiosensitivity and reduced tumor burden in esophageal cancer by inhibiting phosphorylation of c-Met [137]. Identifying new molecular targets may help improve clinical outcomes in ESCC patients. Since elevated c-Met expression was significantly associated with tumor depth and pathological stage and patient survival was significantly poor, possible prognostic factors for ESCC patients were included in a multifactorial analysis, which showed high c-Met expression as an independent prognostic factor [138].

#### Targeted Wnt/β-catenin pathway

β-Catenin, a core member of the typical Wnt signaling pathway, has recently been found to be aberrantly activated in esophageal cancer progression, metastasis and invasion. NRAGE is a melanoma antigen-encoding gene homolog that interacts with neurotrophin-receptors to encode cancer-related proteins. Recent studies have confirmed that IR may promote NRAGE upregulation, which further triggers β-catenin nuclear protein accumulation, induces ESCC cell proliferation and cell cycle rearrangement, and then stimulates procancer activity [139]. Therefore, targeting the Wnt/β-catenin pathway is expected to result in prolonged OS and PFS in ESCC patients treated with IR, significantly improving prognosis. Ras-association domain family 10 (RASSF10) is a potential biomarker involved in ESCC invasion and metastasis. As a tumor suppressor gene targeting the Wnt/β-catenin pathway, RASSF10 overexpression inactivates this pathway and may exert anti-metastatic functions by blocking EMT and inhibiting ESCC cell proliferation [140]. FH535 is an inhibitor of the Wnt/β-catenin pathway. FH535 treatment decreases β-catenin expression and nuclear translocation in the cytoplasm, impairs DNA double-strand break repair, and reverses the EMT phenotype by increasing E-cadherin expression, enhancing the radiosensitivity of esophageal cancer cells [141]. SOX17, a negative regulator in the Wnt pathway, represses the expression of its downstream effector MALAT1 at the transcriptional level and decrease the level of HIF-1α upregulation by targeting miR-199a, thereby enhancing ESCC radiosensitivity [142]. Paired-like homeodomain transcription factor 2 (PITX2) is a downstream effector of Wnt/β-catenin signaling, and inhibition of PITX2 expression and knockdown of its levels triggered more apoptosis, which in turn, significantly enhanced the sensitivity of ESCC cells to IR and cisplatin but appeared to be unrelated to EMT [143]. We summarize the key factors or pathways and major mechanisms associated with radiosensitivity by molecular targeted therapies in Table 2.

**Table 2:** Key factor or pathway and major mechanism corresponding to molecular targeted therapies for radiosensitization

Key factor or pathway	Agent	Major mechanism	Tumor type	Year and Reference
EGFR	Erlotinib	-	Locally advanced ESCC	2020 [179]
EGFR-ERK	Cetuximab	G2/M cycle arrest and DNA repair delay	ECA109 and TE-13 cell lines	2023 [180]
EGFR	Icotinib	-	Older adults with unresectable ESCC	2020 [108]
EGFR	Nimotuzumab + cetuximab	-	Locally advanced ESCC	2019 [106]
VEGF, HIF-1α	2-Methoxyestradiol	Inhibited proliferation of ESCC cells	ECA109 cell line	2019 [116]
VEGF, PD-1	Camrelizumab + apatinib	Anti-angiogenic	Advanced ESCC	2020 [123]
c-Met	BPI-9016 M	Inhibited the ATM- and ATR-dependent DNA damage HR recombination repair	ECA109 cell lines	2021 [74]
c-Met	Foretinib	Inhibited proliferation and prompted the G2/M arrest of ESCC cells, delays the DNA damage repair	ECA109 and TE-13 cell lines	2017 [137]
Wnt/β-catenin	RASSF10	Inhibited epithelium-mesenchymal transition	ESCC patients and TE-10, ECA-109 and KYSE-150 cell lines	2022 [140]
PI3K/Akt/mTOR	FAM135B	Promotes cell cycle redistribution	KYSE150, ECA109, TE-13, TE-10, and TE-1 cell lines	2021 [181]
PI3K/Akt/mTOR	UHRF1	Increased ESCC cell apoptosis	The human ESCC, ECA109, and TE-1 cell lines	2021 [132]

### Thymosin alpha-1(Tα1)

Thymosin alpha-1 (Tα1) is an immunomodulator capable of suppressing many tumor-associated immunosuppression by increasing NK cell and dendritic cell activity, shifting Tregs to Th1 cells subsets, inducing the release of Th1-type cytokines such as IL-2, IFN-α; and upregulating MHC I antigen expression in normal and transformed cells, activating cytotoxic T-cells mediated innate and acquired immune responses. Du et al. [144] first demonstrated that in heavily pretreated ESCC patients, SBRT combined with Tα1 treatment promoted achieved its prespecified endpoint with more than 20% of patients having stable metastatic lesions and facilitated better control of non-irradiated-induced metastatic lesions. Unfortunately, no patients achieved complete remission and only 3 patients achieved partial metastatic-lesion response, with a significantly lower response rate than GM-CSF combined with SBRT, this may be related to the intra-tumor characteristics of high frequency mutations [145, 146]. Moreover, Tα1 has been shown to promote apoptosis in MDSCs not only by decreasing the Bcl-2/BAX ratio, but also by downregulating HIF-1α in tumor cells to inhibit blood VEGF production [147]. Therefore, further exploration of Tα1 combined with radiation means to enhance patient immunity is necessary in the future.

### Epigenetic therapy in combination with radiotherapy

#### DNA methylation inhibitors

DNA methylation occurs when methyl groups are added to cytosine residues in cytosine-guanine (CpG) islands at the 5-carbon position. This affects the ability of DNA to coil around histones and results in a condensed heterochromatin conformation, which prevents genes from being transcribed [148]. High promoter methylation of ESCC suppressor genes, such as p16 [149], RASSF5A [150], SULT2B1 [151], SEMA3B [152], PTPN6 [153] and Bin1 [154] can lead to tumor suppressor gene silencing and cancer cell activation and can be used as a predictor of clinical outcome after radical resection in ESCC patients. Aberrant DNA methylation contributes to the development of radioresistance during anticancer therapy; for example, high methylation of death-associated protein kinase (DAPK) [155], CHFR [156] and FGF5 [157] is associated with a diminished response to CRT in ESCC, in which DNA methyltransferases (DNMTs) play an important role, including DNMT1, DNMT3A and DNMT3B. DNMT1 silencing increases the expression of RASSF1A and DAPK in ESCC cells and decreases the methylation of both promoters, thereby inhibiting ESCC cell proliferation and invasion [158]. The DNMT inhibitor RG108 is a non-nucleoside analog designed to target human DNMT1, which lacks the high level of cytotoxicity associated with 5-Aza-dCR and binds to the active site of DNMT but does not affect the methylation status of the centromeric repeats. Yao et al. [159] found that pretreatment with RG108 followed by IR resulted in G2/M arrest and an increased Bax/Bcl-2 ratio in cancer cells and thus increased the apoptosis of esophageal cancer cells, possibly through a complex mechanism such as the TGF-β pathway that increases the radiosensitivity of esophageal cancer cells. Nutlin-3, a murine double min 2 (MDM2) small molecule inhibitor, upregulated tumor suppressor gene p53 and RB levels in ESCC cells, thereby inhibiting the expression of the three isoforms of DNMT, and reduce methylation levels of multiple tumor suppressor genes, and increased radiosensitivity of ESCC [160].

#### Histone modification inhibitors

**Fig. 3:**
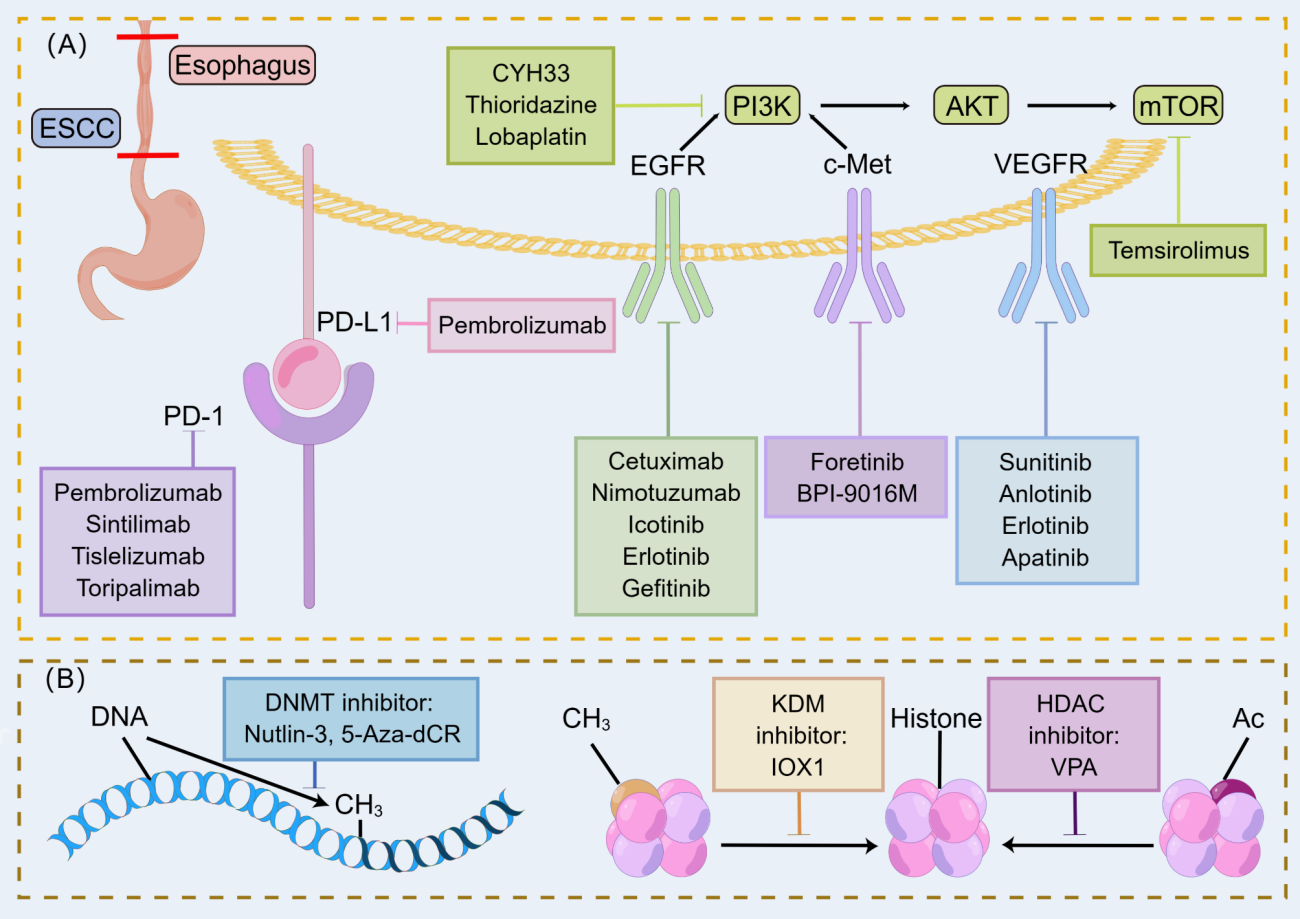
Potential therapeutic targets and agents to reverse ESCC radioresistance. (A) Potential therapeutic agents targeting key pathways. (B) Potential agents for epigenetic therapy. DNMT: DNA methyltransferase; KDM: Histone lysine demethylase; HDAC: Histone deacetylase; VPA: Valproic acid

Histone modifications include acetylation, methylation, phosphorylation, and ubiquitination, with methylation and acetylation being more successful in current ESCC epigenetic therapies. Acetylation of the histone lysine tail neutralizes the positive charge of the lysine, resulting in a weakened electrostatic interaction between the histone and negatively charged DNA. When this acetylation occurs in enhancer and promoter regions, it contributes to a more open true chromatin conformation that is readily bound by the cell’s transcriptional machinery. Histone deacetylase (HDAC) leads to transcriptional silencing, which, together with lysine acetyltransferase (KAT), is a competing enzyme involved in histone lysine acetylation and transcriptional regulation, inhibition of HDAC further leading to chromosome instability, cell cycle arrest and induction of DNA DSBs, which occurs simultaneously with inhibition of protein fingers involved in DNA damage repair; thus, the use of HDAC inhibitors is the key to inhibiting the role of HDAC in radiosensitization. Valproic acid (VPA) induces high acetylation of histones H3, H4 and Ku70 and apoptosis and prolongs IR-induced DSBs by inducing downregulation of the DNA repair protein Rad51 to inhibit HR in the DSB repair pathway in ESCC, thereby enhancing radiation-induced cytotoxicity in human ESCC cells [161]. Panobinostat is a novel HDAC inhibitor that inhibits ESCC cell proliferation mainly by significantly inhibiting TP53 expression while increasing p21 and decreasing cell cycle arrest induced by cyclin D1 expression [162]. Two related studies showed that cyclic hydroxamic-acid-containing peptide 31 (CHAP31) induced apoptosis in ESCC cells by inducing cleavage of caspase 9 and upregulation of the Bax/Bcl-2 ratio, sensitized ESCC cells to carbon ion RT and inhibited the expression of DNA repair-related genes [163, 164]. In addition, activation or inhibition of methylation on histone lysine residues has been shown to act synergistically with acetylation and other modifications, and histone methylation in the ESCC immunohistochemical expression of markers included dimethylated histone 4 arginine 3 (H4R3diMe), dimethylated histone 3 lysine 4 (H3K4diMe) and trimethylated histone 3 lysine 27 (H3K27triMe) [165]. Multifactorial analysis revealed that the expression of H3K27triMe was an independent indicator of prognosis in patients with early ESCC [165]. We summarize the potential therapeutic targets and agents to reverse ESCC radioresistance in Fig. 3.

#### Regulating the expression of non-coding ribonucleic acid

MiRNAs contain 19–24 nucleotides and are derived from cleaved and processed pri-miRNAs. MiRNAs regulate ESCC cell survival and invasion through at least four pathways. (i) Targeting tumor suppressor genes or related proteins. For example, the expression level of miR-21 is increased in radioresistance compared with radiation-sensitive patients. PTEN is a tumor suppressor gene located on chromosome 10q23, so miR-21 enhanced the radioresistance of ESCC by downregulating PTEN [166]. MiR-338-5p [167] can be targeted to survivin to enhance the sensitivity of ESCC to radiation therapy. Kinesin superfamily protein 22 (KIF22) is a target of miR-122 and is highly expressed in ESCC tissues and cancer cell lines. Enhanced miR-122 expression can downregulate KIF22 as a way to promote apoptosis and G0/G1 phase arrest in ESCC cells and significantly inhibit the malignant progression of ESCC cells by suppressing the ability of EMT to inhibit ESCC proliferation, migration and invasion [168]. (ii) Targeting signaling pathways. For example, in small extracellular vesicles derived from dying human ESCC cells after IR, upregulation of miR-26b-5p enhanced immunosuppression by activating the PI3K/AKT signaling pathway to activate MDSC expansion and function [96]. Therefore, knockdown or inhibition of miR-26b-5p may be a potential target to inhibit MDSC recruitment. (iii) Targeting histone-modifying enzymes. For example, miR-199-5p can directly target the expression and function of the deacetylase Sirt1, and knocking out miR-199-5p enhances the cleavage of CD44 and promotes the translocation of CD44ICD to the nucleus [16]. (iv) Regulation of stem cell gene expression. Lu et al. [16] showed that the miR-199-5p/Sirt1 signaling pathway regulates the stemness of ESCC stem cells by regulating the expression of stem cell genes and therefore plays a key role in tumor formation and invasion.

LncRNA, an RNA longer than 200 nt, likewise enhances the radiosensitivity of ESCC cells after RT through at least four pathways. (i) Directing RNA-binding proteins. For example, RECK is a tumor suppressor protein that inhibits the invasion and metastasis of cancer cell lines and tumor angiogenesis [169]. LncRNA GAS5 decreases miR-21 expression to enhance the radiosensitivity of ESCC cells after RT by upregulating RECK levels [170]. (ii) Provide a platform for molecular assembly. LncRNA-NORAD (also known as Lnc00657) is abundantly expressed in several eukaryotic cells, interacts with DNA damage repair and DNA replication-associated proteins, assembles topoisomerase complexes to maintain genomic stability and repair corresponding DNA damage [171] and has been shown to function as a tumor suppressor gene in many cancers. Studies have confirmed that DNA damage activates NORAD, which is highly expressed in radiation-resistant ESCC cells [172]. NORAD delays pri-miR-199a1 maturation by inhibiting pri-miR-199a1processing and responds to IR to promote HR repair [172]. (iii) Inhibiting molecular signaling. NORAD knockdown reduces the efficiency of HR by inhibiting ATR/Chk1 signaling and inhibits DNA repair components, including ATM, ATR, Chk1, and Chk2, thereby enhancing the therapeutic effect of IR on ESCC [172]. (iv) Play a molecularly induced role. NORAD knockdown enhances the effect of the combination of the immune checkpoint inhibitor PD-1 and RT in the treatment of ESCC [172], but only a small number of ESCC patients have benefited. Table 3 summarizes the effect of targeted non-coding RNA in combination with IR for ESCC.

**Table 3:** The effect of non-coding RNA targeted therapy combined with IR in the treatment of ESCC

Non-coding RNA	Target	The effect	Reference
miR-26b-5p	PTEN/PI3K/AKT signaling pathway	Radioresistance	[96]
miR-205	PTEN/PI3K/AKT signaling pathway	Radioresistance	[182]
miR-519	PI3K/AKT/mTOR signaling pathway	Radiosensitivity	[134]
miR-4443	PTPJR	Radioresistance	[183]
miR-181a-2-3p	RAD17/Chk2 signaling pathway	Radiosensitivity	[176]
miR-193a-3p	PSEN1	Radioresistance	[184]
miR-199a-5p	EEPD1/ATR/Chk1 signaling pathway	Radiosensitivity	[172]
miR-301a	WNT1/β-catenin signaling pathway	Radiosensitivity	[45]
miR-338-5p	survivin	Radiosensitivity	[167]
miR-339-5p	CDC25A	Radiosensitivity	[185]
miR-450a-5p	DUSP10	Radiosensitivity	[186]
LNCRNA FAM201A	miR-101-ATM/mTOR axis	Radioresistance	[187]
LNCRNA MALAT1	Cks1	Radioresistance	[188]
LNCRNA DNM3OS	PDGFβ/ PDGFβR/FOXO1	Radioresistance	[73]
LINC00473	a) miR-497-5p/CDC25A axis b) miR-374a-5p/SPIN axis	Radioresistance	[189, 190]
LINC01004	LINC01004-SPI1 axis	Radioresistance	[86]
circ-100,367	miR-217/Wnt3 signaling pathway	Radioresistance	[191]
circ-0014879	miR-519-3p/CDC25A axis	Radioresistance	[192]
circVRK1	miR-624-3p/PTEN/PI3K/AKT signaling pathway	Radioresistance	[44]

### Overcoming cancer hypoxia

Radioisotopes have long been used for in vivo RT of cancer patients, in which high-energy X-rays, electron beams or proton beams can destroy the DNA structure and thus induce apoptosis in tumor cells. However, limited to intratumor hypoxia, its efficacy is drastically reduced by severe side effects [173]. The current cutting-edge bioengineering nanotechnology aims to develop a decahedral nanoenzyme, a palladium decahedral enzyme modified with folic acid that enables active targeting of iodine-125 (^125^I) to the tumor region, exhibiting good biocompatibility: on the one hand, it catalyzes the conversion of intracellular hydrogen peroxide to oxygen, alleviating hypoxia in TME [174]; on the other hand, it supports the production of free radicals and induces apoptosis in esophageal cancer cells by depositing radiation therapy energy in tumor tissues through palladium [174, 175]. Similar to decahedral nanoenzymes, multifunctional graphdiyne-cerium oxide nanozymes can firmly anchor and disperse the nanocomposite formed by CeO_2_-nanoparticles that exhibit excellent peroxidase activity during the breakdown of hydrogen peroxide [176]. As mentioned previously, non-coding RNAs such as miRNAs play a key role in scavenging ROS, abnormal cellular autophagy and EMT. Nanoceria can facilitate nanoscale delivery of miRNA and prevent endogenous ribonuclease degradation [176]. Meanwhile, the enzyme could enhance intracellular radiation energy deposition to improve targeting efficiency and significantly alleviate ESCC cell hypoxia by reducing HIF-1α to enhance esophageal cancer radiosensitivity [176]. Yao et al. [177] further demonstrated that tumor oxygenating nanoliposomes synergize with HIF-1 inhibitors by synergizing with endogenous oxygen production, enhancing ^125^I proximity radiation targeting implantation and inhibiting tumor growth. We compared the strengths and weaknesses of different approaches for reversing ESCC radioresistance as well as directions for future study in detail in Table 4.

**Table 4:** The strengths and weaknesses of the different approaches to reverse ESCC radioresistance and future directions

Approaches	Strengths	Weaknesses	Future directions
Immunotherapy	1) It is suitable for ESCC patients with high recurrence rate after surgery or neoadjuvant therapy [193]; 2) For metastatic ESCC, treatment combing RT to immunochemotherapy is important for symptom improvement and survival prolongation [194].	1) The evidence of using ICIs in the neoadjuvant setting is lacking; 2) PD-1 is heterogeneously expressed in tumors; 3) The consensus about the PD-L1 assays, OS, PFS and cut-offs are lacking.	1) Validate reliable predictive biomarkers, and then combining biomarkers and intelligent immunotherapy; 2) Reduction in toxicity associated with combination therapy.
Molecular targeted therapy	1) Provide some benefit in an adjuvant setting in patients with locally advanced ESCC to prevent or delay relapse; 2) Dual target (such as EGFR and Wee1) may enhance therapeutic effect [195].	1) It has failed to demonstrate significantly improved OS in clinical trials for patients with recurrent or metastatic ESCC; 2) Intratumorally heterogeneity.	1) Identifying populations susceptible to inhibition by specific molecules; 2) Drugs development based on signaling crosstalk.
Epigenetic modification inhibitors	1) DNMTi and HDACi can be used in combination with antitumor drugs, improving their efficacy and reducing the toxic effects; 2) NcRNAs may serve as some novel prognostic biomarkers; 3) The specific interactions between ncRNAs and ferroptosis [196]; 4) Abnormal expression of circRNA could serve as a warning indicator of early tumor diagnosis [197]; 5) Nanozymes promote endogenous H_2_O_2_ catabolism and miRNA delivery, enable efficient effect of RT [176, 198].	1) Using a single inhibitor alone is not enough to fundamentally change the prognosis of cancer patients, and side effects cannot be avoided; 2) The application in clinical settings has been hampered by the lack of specificity, delivery method, and tolerability; 3) The functions of aberrations in histone PTMs machinery remain largely unclear [199].	1) Reducing the toxic effects of drugs; 2) Exploring the relationship and combined efficacy of natural products and DNMTi; 3) Finding a proper approach for the delivery of miRNA to the target area with effectiveness and without being degraded by endogenous RNases [200]; 4) Exploring the functions and molecular mechanisms of ncRNAs on ferroptosis.

ICIs: immune-checkpoint inhibitors; DNMTi: DNA methyltransferase inhibitor; HDACi: histone deacetylase inhibitor; PTMs: post-translational modifications; NcRNAs: non-coding RNAs; RNases: ribonucleases

## Conclusion and future perspectives

Designing novel therapeutic options for esophageal cancer is particularly difficult due to the high degree of molecular heterogeneity and the absence of proven biomarkers for early detection of malignancy. Despite a large body of research, the molecular basis of response to radiotherapy in ESCC patients is not fully understood. This review attempts to dissect the major molecular mechanisms involved in ESCC radioresistance, including CSCs, enhanced DNA damage repair capacity, enhanced ROS scavenging, EMT, aberrant programmed cell death, and TME, and attempts to summarize the latest therapeutic strategies. These analyses depict genomic dysregulation and microenvironmental alterations that may lend themselves to specific molecular suppression in ESCC, making a small contribution to the development of precision medicine. Differentiation clusters can be used as surface markers of ESCC CSCs and inhibit the maintenance of tumor stemness by suppressing their expression, making them independent prognostic factors and promising therapeutic targets for ESCC. Our laboratory has been exploring pathways and targets to reverse ESCC radioresistance, and identify RSK4 as a key factor driving CSC characteristics and ESCC radioresistance [11]. In the future, we will continue to explore ESCC CSC-associated protein kinases and their effects on radiation therapy to find targeted drugs for CSCs.

Moreover, ferroptosis is a hot spot in current research. As Nrf2 can bind to ARE in ESCC cancer cells, prompting the cells to adapt to oxidative stress. binding of Nrf2 to SLC7A11 can reduce the cellular ROS production and lipid peroxidation level, which is a novel radioresistance-inducing mechanism and therapeutic target. This suggests that we can design radiosensitization strategies for ESCC patients starting from the Nrf2/SLC7A11/ferroptosis axis [58]. Like ferroptosis, autophagy also serves as a mode of programmed death, and more therapeutic options could be designed by targeting mTOR in the future [178].

Currently, relevant targeted agents for esophageal cancer are a hot research topic, and the era of immunotherapy for ESCC treatment started with the FDA approval of pembrolizumab for advanced ESCC patients in 2019. Several potential biomarkers, including tumor PD-L1 expression, mismatch repair defects, non-coding RNA, and thymidine have been validated in clinical trials. Targeted therapies can prolong patients’ lives, but are prone to other complications because of the many crossovers between drug-regulated signaling pathways. And drug response varies from population to population, which will increase the cost of treatment and monitoring. There is an urgent need to design combination therapy regimens for further preclinical and clinical trials in the future, attention also needs to be paid to severe toxicity from combination therapy.

## Data Availability

not applicable.
